# SPC24 boosts tumor progression and correlates with immune infiltrates in pancreatic adenocarcinoma

**DOI:** 10.3389/fonc.2026.1558646

**Published:** 2026-04-23

**Authors:** Wenhui Chen, Xianyu Huang, Jiaxin Liu, Yonghui Liao, Dingwen Zhong

**Affiliations:** Department of Hepatobiliary and Pancreatic Surgery, The Affliated Ganzhou Hospital of Nanchang University (Ganzhou People's Hospital), Ganzhou, Jiangxi, China

**Keywords:** Immune escape, mitosis, pancreatic ductal adenocarcinoma (PDAC), single cell sequencing, SPC24, tumor immune microenvironment

## Abstract

**Background:**

Pancreatic ductal adenocarcinoma (PDAC) is known for its highly aggressive nature, difficult diagnosis, and resistance to treatment, resulting in a long-term five-year survival rate of less than 10%. This study focuses on the expression and function of mitotic spindle core component SPC24 in PDAC, systematically revealing its important role in tumor progression and prognosis.

**Methods:**

Based on TCGA, GEO, and single-cell RNA sequencing data, SPC24 was significantly overexpressed in PDAC tissues and malignant cell subsets, and its expression level was significantly negatively correlated with overall survival (OS) and progression-free survival (PFS).

**Results:**

Bioinformatics analysis showed that SPC24-related genes were mainly enriched in cell cycle regulation, chromosome segregation and spindle assembly pathways, suggesting that SPC 24-related genes play a driving role in genomic instability and tumor clone evolution. Immunoinfiltration analysis revealed that high SPC24 expression was associated with immunosuppressive microenvironment features, such as increased M2 tumor-associated macrophages and regulatory T cells, and decreased CD8 + T cells and NK cells. Single-cell communication analysis further suggests that SPC24 may facilitate immune escape by regulating ligand-receptor interactions between tumor cells and immune cells in TME. In addition, *in vitro* and nude mice xenograft models verified the promotion of SPC24 on PDAC cell proliferation, migration and tumor growth. The prognostic risk model based on SPC24 showed good predictive performance and had potential clinical application value.

**Conclusion:**

SPC24, as an important oncogenic factor and prognostic marker of PDAC, promotes tumor progression and treatment resistance by regulating mitotic abnormalities, metabolic reprogramming and immune microenvironment, and is worthy of being a candidate molecule for new strategies and diagnostic monitoring of targeted therapy. Future studies are needed to further explore its molecular mechanisms and potential for combination therapy to improve clinical outcomes in patients with PDAC.

## 1 Introduction

### 1.1 Pancreatic cancer: serious challenges and urgent escape from research

With a five-year survival rate of less than 10%, pancreatic ductal adenocarcinoma (PDAC) is a highly deadly and aggressive malignant tumor that ranks in the seventh largest cause of cancer-related deaths globally, accounting for 432,242 deaths in 2018. Although five-year survival rates have increased from 2.5% to around 10% over the last 30 years due to advancements in detection and therapy, they are still significantly lower than those of other prevalent cancer types. Biological aggressiveness (early metastasis to adjacent vital organs, high tendency to metastasize), diagnostic challenges (80%-85% of patients are at advanced stages at diagnosis, lack of early specific symptoms), and treatment resistance (chemotherapy resistance due to complex tumor microenvironment, only 15-20% of patients are eligible for surgery) are some of the factors contributing to the incredibly low survival rate of PDAC. This 40-year survival standstill emphasizes PDAC as a deadly, clinically unresolved problem (1–5). The following important features of PDAC are mostly responsible for this dire prognosis: It is challenging to make an early occult diagnosis: Early-stage PDAC often exhibits no particular symptoms; after clinical signs show up, the tumor is frequently progressed and may have distant metastases or local invasion; Extremely invasive and metastatic: PDAC cells may easily infiltrate adjacent tissues, have a high rate of infiltration, and spread quickly via the bloodstream or lymphatic system to distant organs such the liver, lung, and peritoneum, making therapy extremely challenging; Tumor microenvironment complexity: PDAC tumor microenvironment (TME) is rich in hypoxia, immunosuppressive cells (including bone marrow-derived suppressor cells (MDSCs) and tumor-associated macrophages (TAMs), and fibrogenic stroma, all of which work together to create a potent immune escape barrier and drug resistance mechanism; overall resistance to conventional therapy: PDAC is less responsive to radiation and chemotherapy (including gemcitabine), and the majority of patients either relapse or worsen shortly after starting conventional treatment. The medical community finds it difficult to address PDAC because of these tough biological features. To enhance patient survival and prognosis, a thorough examination of the pathophysiology of PDAC, the discovery of novel prognostic biomarkers, and the investigation of novel treatment targets are thus crucial. The development of multi-omics technologies, such as single-cell sequencing, transcriptomics, proteomics, and genomics, has given us hitherto unheard-of chances to completely comprehend the complexity of cancer and identify novel treatment targets (6, 7).

### 1.2 Mitotic spindles play a key role in cell proliferation and carcinogenesis

An even distribution of genetic material among daughter cells is guaranteed by the very accurate and strictly controlled process of mitosis. For mitotic precision to be guaranteed, spindles must be assembled and operated correctly. Microtubule and microtubule-associated protein make up the majority of the spindle. Its primary job is to identify and distinguish between sister chromatids (8). Important processes in spindle assembly include microtubule nucleation, polarization, and dynamic instability control. The primary microtubule nucleation center in cells, the γ-tubulin complex (γ-TuRC), is in charge of starting microtubule development at the two polar bodies of cells. Defects in spindle structure, including spindle asymmetry and chromosomal segregation mistakes (aneuploidy), are often caused by abnormal γ-TuRC composition and function. These issues play a significant role in the development of tumors and the conversion of cells into cancer cells (9, 10). Numerous investigations have shown that spindle assembly and function-related gene mutations or aberrant expression are prevalent in various malignancies and strongly correlate with patient prognosis. For instance, the Aurora kinase family (AURKA, AURKB) and its downstream effectors (e.g., PLK1, CDC20) are often overexpressed in a range of malignancies, are linked to a bad prognosis, and play important roles in the control of spindle checkpoint (SAC). Furthermore, tumor invasion and metastasis are tightly linked to aberrant expression of proteins like KIF2C and KIF20A that regulate microtubule dynamics (11, 12).

### 1.3 SPC24: important gamma-TuRC constituents and their possible connection to cancer

One of the stable subunits of γ-TuRC is SPC24 (Spindle pole protein E), which combined with SPC25 (Spindle pole protein F) make up the core structure of γ-TuRC. The efficient nucleation of microtubules in centrosomes and non-centrosomes depends critically on the presence and function of the γ-TuRC complex. The stability and effective nucleation of γ-TuRC are dependent on the structural integrity and proper cellular localization of SPC24. More and more data in recent years have shown that SPC24 is not only a structural protein but also has a significant impact on the development of cancer and the control of the cell cycle. In some cancer types, it has been proposed that the expression level of SPC24 is connected to tumor invasion, metastasis, and proliferation (13). SPC24 expression is strongly correlated with immune cell infiltration, immune molecules, and tumor mutation burden (TMB); the miR-501-3p/SPC24 axis affects cell proliferation; the expression level of SPC24 is significantly correlated with TNM stage; and elevated SPC24 indicates a worse prognosis. SPC24 is significantly upregulated in a variety of cancers, particularly renal clear cell carcinoma (KIRC) and renal papillary cell carcinoma (KIRP). Apoptosis, invasion, and migration. SPC24 regulates the PI3K/AKT pathway, which contributes to the oncogenic role of breast cancer development. CDK1 kinase activity controls the connection between SPC24/25 dimer and CENP-TW dimer during cell division (14–16).

### 1.4 The innovation and goal of this study

This research will concentrate on SPC24 function in PDAC because of the poor prognosis of PDAC and the possible involvement of SPC24 in other malignancies. We will use multidimensional bioinformatics analysis and prognostic value evaluation to provide a detailed description of SPC24’s function in PDAC. We will examine the connection between SPC24 mRNA and protein expression levels and patient survival (OS and PFS) using a large public PDAC genomic and clinical database like TCGA (The Cancer Genome Atlas). Statistical techniques such as Kaplan-Meier survival analysis and Cox proportional hazards models will be used to evaluate SPC24’s potential as an independent prognostic factor. Profiling gene expression and analyzing pathways: Genes that were strongly correlated with SPC24 expression levels were found using differential expression analysis. Additionally, key signaling pathways that SPC24 could be engaged in were investigated using pathway enrichment analysis tools including Gene Ontology and KEGG (Kyoto Encyclopedia of Genes and Genomes). This helps in elucidating the molecular mechanism by which SPC24 operates in PDAC. Examination of the immune infiltration and tumor microenvironment: The link between the tumor microenvironment and the degree of infiltration and the immunosuppression/activation state of immune cells (such as T cells, B cells, macrophages, and NK cells) in the PDAC tumor microenvironment was examined using the QuanTIseq method. This helps us comprehend SPC24’s possible function in tumor immune escape. Comprehensive examination of data from single-cell RNA sequencing (scRNA-seq): SPC24 expression varies across PDAC cell subsets: Using the published PDAC scRNA-seq dataset, the heterogeneous expression of SPC24 in stromal cells, immune cells, tumor cells, and other cell types was thoroughly documented. SPC24-expressed tumor cell subgroups that may be linked to treatment resistance, metastasis, or invasiveness will be identified. SPC24 and tumor cell function are correlated: To comprehend the possible involvement of SPC24 in tumor cell proliferation, differentiation, migration, immune evasion, and contact with other cells, single-cell cell-cell communication analysis is used.

## 2 Materials and methods

### 2.1 Data sources and pre-processing

TCGA database analysis: Gene expression data (RNA-seq V2 RSEM), clinical information and survival data for pancreatic cancer (PAAD) were downloaded from The Cancer Genome Atlas (TCGA) database. The data download date is August 15, 2025. R language (v4.3.3) and its related packages (such as TCG Abiolinks, DESeq2, survival, glmnet, ComplexHeatmap, ggplot2) were used for differentially expressed gene analysis (DEGs), weighted gene co-expression network analysis (WGCNA), prognostic model construction (one-way Cox regression, LASSO regression, multi-way Cox regression), nomogram construction, gene set enrichment analysis (GSEA) and immune microenvironment analysis (immune microenvironment analysis). Expression data for the SPC24 gene were obtained through the TCGA-PAAD project.

GEO Database Verification: Dataset GSE16515 (containing PDAC tissue samples and normal control samples) was downloaded from the Gene Expression Omnibus (GEO) database to verify differences in expression of SPC24. The data download date is August 30, 2025. Differential expression analysis was performed using R language (v4.3.3) and limma package.

Single-cell sequencing data analysis: Publicly available single-cell RNA sequencing data (GSE155698) was used to analyze cell composition and SPC24 expression in different cell types of PDAC patients. Seurat (v4.3.0) and harmony packages were used for data preprocessing, cell subset identification, expression analysis of SPC24, and pathway enrichment analysis (ReactomeGSA).

Mitotic spindle formation disorder genes Source: Mitotic spindle formation disorder related genes (MSDs) were obtained from the literature list: After removing duplicate genes, there were 36 MSDs in total (17).

### 2.2 Bioinformatics analysis

This study integrates multiple bioinformatics approaches to comprehensively investigate the prognostic value of SPC24 in pancreatic cancer (PDAC) and its underlying molecular mechanisms. First, we performed differentially expressed genes (DEGs) analysis on gene expression data from PDAC samples and normal control samples from the TCGA database using the DESeq2R package to identify genes with significantly altered expression in PDAC. Then, we used WGCNA (Weighted Gene Coexpression Network Analysis) R package to construct gene coexpression network and identify gene modules significantly associated with it according to MSDs score (tumor microenvironment-related gene score). Next, we identified candidate genes by performing intersection analysis of DEGs with genes in gene modules identified by WGCNA as significantly associated with MSDs scores. Subsequently, Gene Ontology (GO) and Kyoto Encyclopedia of Genes and Genomes (KEGG) pathway enrichment analysis were performed on these candidate genes using clusterProfiler R package to reveal key biological processes and signaling pathways related to the occurrence and development of PDAC.

To assess the prognostic value of SPC24, we used Cox proportional hazards models for prognostic gene identification and model construction. Specifically, genes associated with overall survival (OS) of PDAC patients were initially screened by univariate Cox regression analysis, and then LASSO (Least Absolute Shrinkage and Selection Operator) regression and multivariate Cox regression analysis were combined to identify independent prognostic factors and construct a risk score model capable of predicting patient prognosis. Based on the constructed risk score model, we further constructed nomograms and assessed the predictive performance and accuracy of the model through calibration curves and receiver operating characteristic (ROC) curves.

In addition, to further understand the role of risk models and related genes in biological function, we performed Gene Set Enrichment Analysis (GSEA) to explore the enrichment of prognostic genes and different risk groups in specific biological pathways. In terms of tumor microenvironment, we used the immunedeconv R package to evaluate the infiltration level of different immune cells in PDAC tumor tissues by integrating multiple algorithms and analyzing the correlation between the degree of infiltration of these immune cells and patient risk scores and the expression level of key prognostic genes. Finally, in order to further explore the potential of SPC24 in clinical applications.

This study also provided an in-depth analysis of the publicly available PDAC single-cell RNA sequencing (scRNA-seq) dataset. Raw single-cell data were preprocessed, clustered, and dimensionalized (e.g., UMAP) using Seurat R packages to identify different cell types and subpopulations. Through marker gene annotation, we identified key cell groups such as tumor cells, immune cells and stromal cells. On this basis, we accurately mapped the heterogeneity of SPC24 expression across different cell types and tumor cell subsets and processed multiple datasets using algorithms such as Harmony to ensure robustness of the results. In addition, we performed pathway enrichment analysis on a subset of tumor cells that overexpress SPC24 using tools such as ReactomeGSA to reveal key biological processes they may be involved in, such as cell cycle regulation, mitosis, and signaling patterns with other cells.

### 2.3 Cell experiments

Cell proliferation assay: Cell proliferation ability was evaluated by CCK-8 and colony formation assay. CCK-8 was tested using Cell Counting Kit-8 (K1080, APExBIO, USA). For colony formation experiments, 500 cells were seeded per well of a six-well plate. After 24 hours of incubation, treatment was repeated every five days with increasing concentrations of acetazolamide (1 to 1000 µM). After two weeks of culture, cells were washed with PBS and stained with crystal violet. Finally, colonies with more than 50 cells per well were counted and analyzed quantitatively.

Cell migration experiments: Cell migration ability was assessed by Boyden chambers (Transwells). PDAC cell suspensions (200 µL) silenced or overexpressed by SPC24 were placed in the upper chamber of a 24-well Boyden chamber (3450, Corning, USA) with an 8-µm pore size. The lower compartment contains medium containing 20% fetal bovine serum (FBS) as a chemokine. Cell migration time was set at 24 hours for Panc-1 cells and 48 hours for AsPC-1 cells. Cells migrating to the underside of the membrane were stained with crystal violet. The average number of migrating cells per chamber was assessed by counting.

### 2.4 Animal experiments

To verify the effect of SPC24 on PDAC tumor growth and proliferation *in vivo*, we constructed a mouse subcutaneous tumorigenesis model. We purchased 20 male BALB/c nude mice aged 4 weeks from Shanghai Slack Laboratory Animal Co., Ltd. Each group of 5 mice was injected subcutaneously with PATU-8988 cells overexpressing SPC24, MIA PaCa-2 cells knocking down SPC24, or their respective control cells. At 21 days after injection, mice were euthanized by cervical dislocation and tumor tissue was collected and weighed. All animal experiments were approved by the Institutional Animal Care and Use Committee (IACUC) of the Affiliated Ganzhou Hospital of Nanchang University. In addition, the ARRIVE guidelines (http://arriveguidelines.org) were strictly followed in this animal experiment.

## 3 Results

### 3.1 Differential expression gene analysis

**Figure 1:**
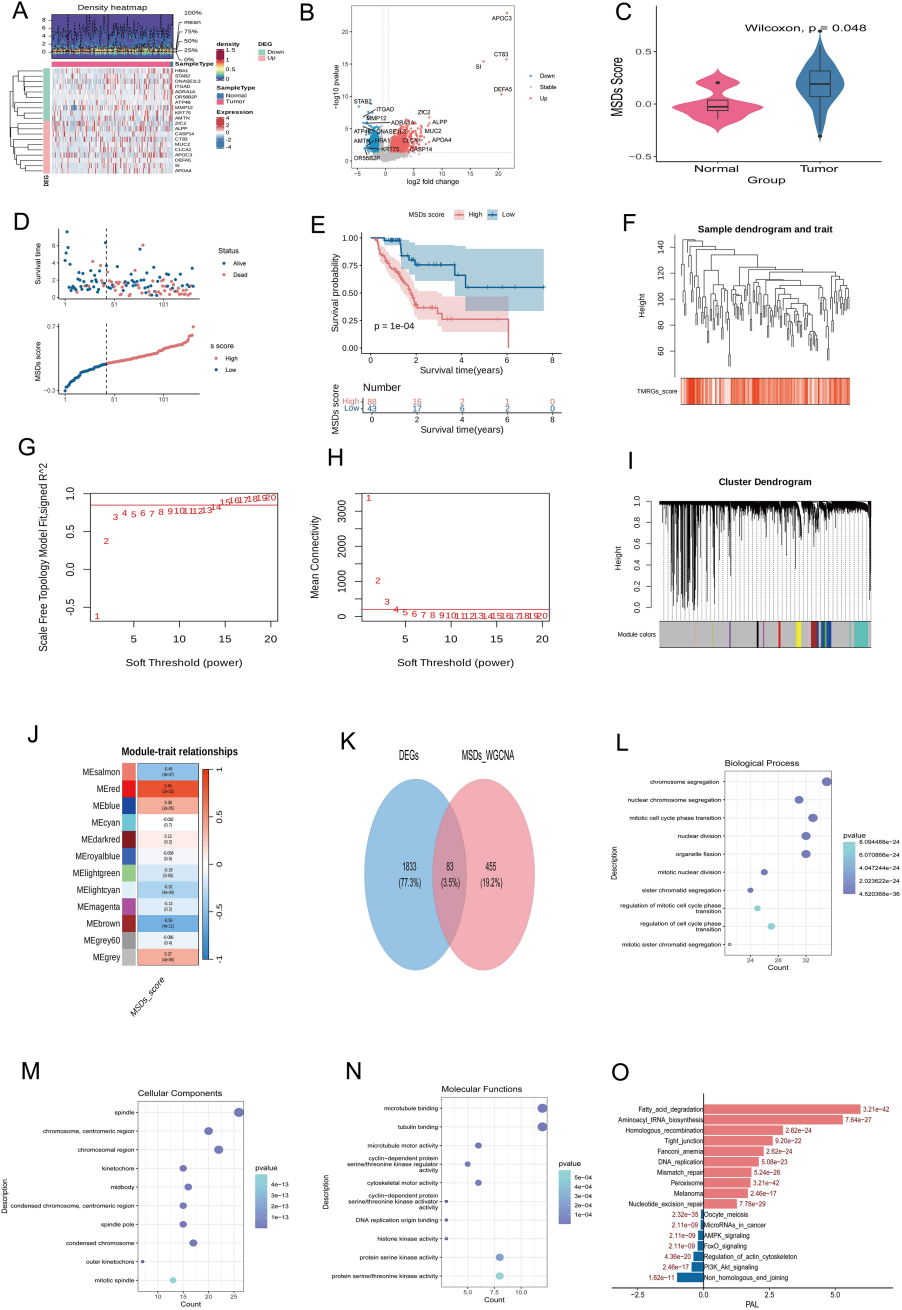
**(A)** Volcano diagram of gene distribution difference between PDAC sample and Control sample, abscissa is Log2FC, ordinate is-Log10 (P.adj), each point represents a gene; **(B)** Heat map of differential gene expression, middle comment bar, red represents PDAC sample, blue represents Normal sample; color of upper density heat map represents gene expression density of each sample, the redder the color, the higher the density; **(C)** Comparison of MSDs scores among different groups of samples in training set, box plot in middle of violin plot shows median and quartile of data; **(D)** The upper figure is the survival status distribution diagram, with the abscissa representing each patient and the ordinate representing survival time; the lower figure is the MSDs score distribution diagram, with the abscissa representing each patient and the ordinate representing MSDs score; **(E)** is the K-M curve, with the abscissa representing survival time and the ordinate representing survival probability; the lower table represents the survival number of the two groups at each time point; **(F)** is the sample cluster diagram, and the lower heat map block represents the MSDs score of each sample; **(G, H)** are the soft threshold values determined according to the relationship graph between the soft threshold values and the scale-free network evaluation coefficient R2 and the average connectivity to construct the scale-free network, the abscissa is the soft threshold values, and the ordinate is the corresponding scale-free network evaluation coefficient and average connectivity, respectively. **(I)** is the identification of gene modules using hierarchical clustering and dynamic shear tree; **(J)** is the correlation heat map between gene modules and MSDs scores, the color blocks in the heat map, red represents positive correlation, blue represents negative correlation; **(K)** is the identification of candidate genes, blue circles represent DEGs, red circles represent genes related to MSDs scores, and the overlapping part in the middle represents genes in two gene sets at the same time, i.e. candidate genes; **(L–N)** are GO enrichment analysis, the size of the dot represents the number of enriched genes, the larger the dot represents the more enriched genes; the color of the dot represents the p value, the smaller the value, the darker the color, the more significant the enriched pathway, the ordinate represents the name of the enriched pathway; **(O)** is the candidate gene KEGG pathway activation level analysis, the horizontal axis is PAL score, PAL greater than 0 is active state, PAL less than 0 is inhibition state, and the vertical axis is KEGG pathway name.

In order to identify the differential expression genes (DEGs) between different sample groups and provide the basis for subsequent functional mining, we first used the R package “DESeq2” to analyze the differential expression of PDAC samples and Control samples. The screening criteria are adjusted p-value (p. adj) < 0.05 and|log2FC|>0.5. As shown in **Figure 1A**, in the TCGA training set, a total of 5,485 DEGs were identified between the PDAC sample and the Control sample, of which 2,979 genes were upregulated and 2,506 genes were downregulated. The volcano diagram (**Figure 1B**) visually shows the distribution of these differential genes (**Figure 1A**).

### 3.2 Comparison of MSDs scores between different groups of samples

In order to evaluate the difference in MSDs scores between PDAC samples and Control samples, we used the R package “GSVA” to calculate the MSDs scores of all samples based on gene expression. Compare the MSDs scores of the two groups of samples through the Wilcoxon test, and visualize them using ggplot2 (**Figure 1C**). The results showed that the MSDs score of the PDAC sample was significantly lower than that of the Control sample (p = 0.048), suggesting that the MSDs score may play a role in the occurrence and development of PDAC.

### 3.3 The relationship between MSDs score and patient prognosis

In order to explore the association between MSDs score and the prognosis of PDAC patients, we used TCGA training to focus on PDAC patients with survival information. According to the best truncation value of the MSDs score, the patients are divided into high MSDs score group and low MSDs score group,

As shown in **Figure 1D**, it can be seen from the figure that the proportion of deaths among patients in the low MSDs scoring group is significantly lower than that of the high MSDs scoring group. In order to quantify this difference in survival, we used the R package “survivminer” to plot the Kaplan-Meier (K-M) survival curves of high and low groupings of patients (**Figure 1E**). The K-M curve shows that the survival probability of patients in the low MSDs scoring group is significantly higher than that of the high MSDs scoring group (p=1e-04), indicating that the MSDs score is an important prognostic indicator. The table below shows the number of survivors in the two groups at each point in time, which further supports this conclusion.

### 3.4 Identification of modular genes and their correlation with MSDs scores

In order to mine the gene modules related to MSDs scoring, we used WGCNA (Weighted Gene Co-expression network analysis) to analyze the TCGA training set. Outlier samples were eliminated through sample clustering (**Figure 1F**), and 268 PDAC samples were retained. In order to construct a scale-free network, we determined the optimal soft threshold based on the relationship between the soft threshold and the evaluation coefficient R2 of the scale-free network and the average connectivity (**Figures 1G, H**). **Figure 1G** shows that when the soft threshold value is 6, R2 is close to 0.8, which meets the evaluation coefficient requirements of a scale-free network. **Figure 1H** shows that when the soft threshold is 6, the average connectivity is also relatively high. Therefore, we choose the soft threshold power=6 to build the network. Using the soft threshold power=6, gene modules are identified through hierarchical clustering and dynamic shear trees (**Figure 1I**). A total of 12 gene modules were identified and represented by different colors. Subsequently, we calculated the correlation between each gene module and the MSDs score, and drew a correlation heat map (**Figure 1J**). The results showed that the MEred module had the most significant positive correlation with the MSDs score (cor =0.84, p = 2e-32), and the MEsalmon module had the most significant negative correlation with the MSDs score (cor= -0.45, p = 3e-7). We selected the MEred module (containing 538 genes) with the highest correlation with the MSDs score as the key module, and used the genes as the source of candidate genes.

### 3.5 Identification and functional enrichment analysis of candidate genes

In order to identify candidate genes related to the development of PDAC, we analyzed the intersection of differential expression genes (DEGs) and genes in the MSDs scoring-related module (MEred module). As shown in **Figure 1K**, the Venn diagram shows the intersection of DEGs (1916) and MSDs_WGCNA module genes (538). The results showed that a total of 83 genes exist in these two gene sets at the same time, which is the candidate gene we identified. In order to gain an in-depth understanding of the functions of these candidate genes, we used the “clusterProfiler” package to perform GO (Gene Ontology) and KEGG (Kyoto Encyclopedia of Genes and Genomes) pathway enrichment analysis. GO analysis covers three aspects of biological process (BP), cell composition (CC), and molecular function (MF). GO enrichment analysis: 1) Biological process (BP): As shown in **Figure 1L**, candidate genes are significantly enriched in processes related to cell cycle regulation, including “chromosome segmentation”, “mitotic cell cycle phase transition” and “regulation of mitotic cell cycle phase transition”. The size of the dot represents the number of genes enriched, and the larger the dot, the more genes enriched; the color of the dot represents the p value, and the smaller the value, the darker the color, the more significant the enriched pathway; 2) Cell composition (CC): as shown in **Figure 1M**. It shows that candidate genes are mainly enriched in the nucleus and chromosomal regions, such as “chromosome, centromeric region”, “chromosomal region” and “condensed chromosome, pericentromeric region”; 3) Molecular function (MF): As shown in **Figure 1N**, candidate genes are mainly bound to and stimulated by microtubules in terms of molecular function. Enzyme activity is related, including “microtubule binding”, “cytoskeletal motor activity” and “serine/threonine kinase activity”. Candidate gene KEGG pathway activation level analysis: In order to further evaluate the activation or inhibition status of the KEGG pathway enriched by the candidate gene, we used the OncoboxPD database to calculate the pathway activation level (PAL) score of each pathway. A PAL value greater than 0 indicates pathway activation, and a value less than 0 indicates pathway inhibition. As shown in **Figure 1O**, the pathway enriched by the candidate gene is generally activated in PDAC tumor samples, including Fatty_acid_degradation (fatty acid degradation) (PAL = 3.21 e-42), Aminoacyl_tRNA_biosynthesis (Aminoacyl-tRNA biosynthesis) (PAL = 8.62 e-24), Homologous_recombination(homologousRecombination)(PAL = 9.26e-22),Fanconi_anemia pathway (Fanconi_anemia pathway) (PAL = 2.62e-24), DNA_replication (DNA replication) (PAL = 5.08 e-23), Mismatch_repair (mismatch repair) (PAL = 5.24 e-26), Nucleotide_excision_repair (Nucleotide excision repair) (PAL = 7.78e-29), Oocyte_meiosis(oocyte meiosis) (PAL = 2.32e-30), microRNAs_in_cancer (microRNA in cancer) (PAL = 2.11e-09), AMPK_signaling (AMPK signaling pathway) (PAL = 2.11e-09), Regulation_of_actin_cytoskeleton (actinCytoskeletal regulation) (PAL = 4.36e-20) and Non_homologous_end_joing (non-homologous terminal connection) (PAL = 1.82e-11) and other pathways showed significant activation, suggesting that these pathways may play an important role in the development of PDAC.

### 3.6 Screening and model construction of prognostic-related genes

**Figure 2:**
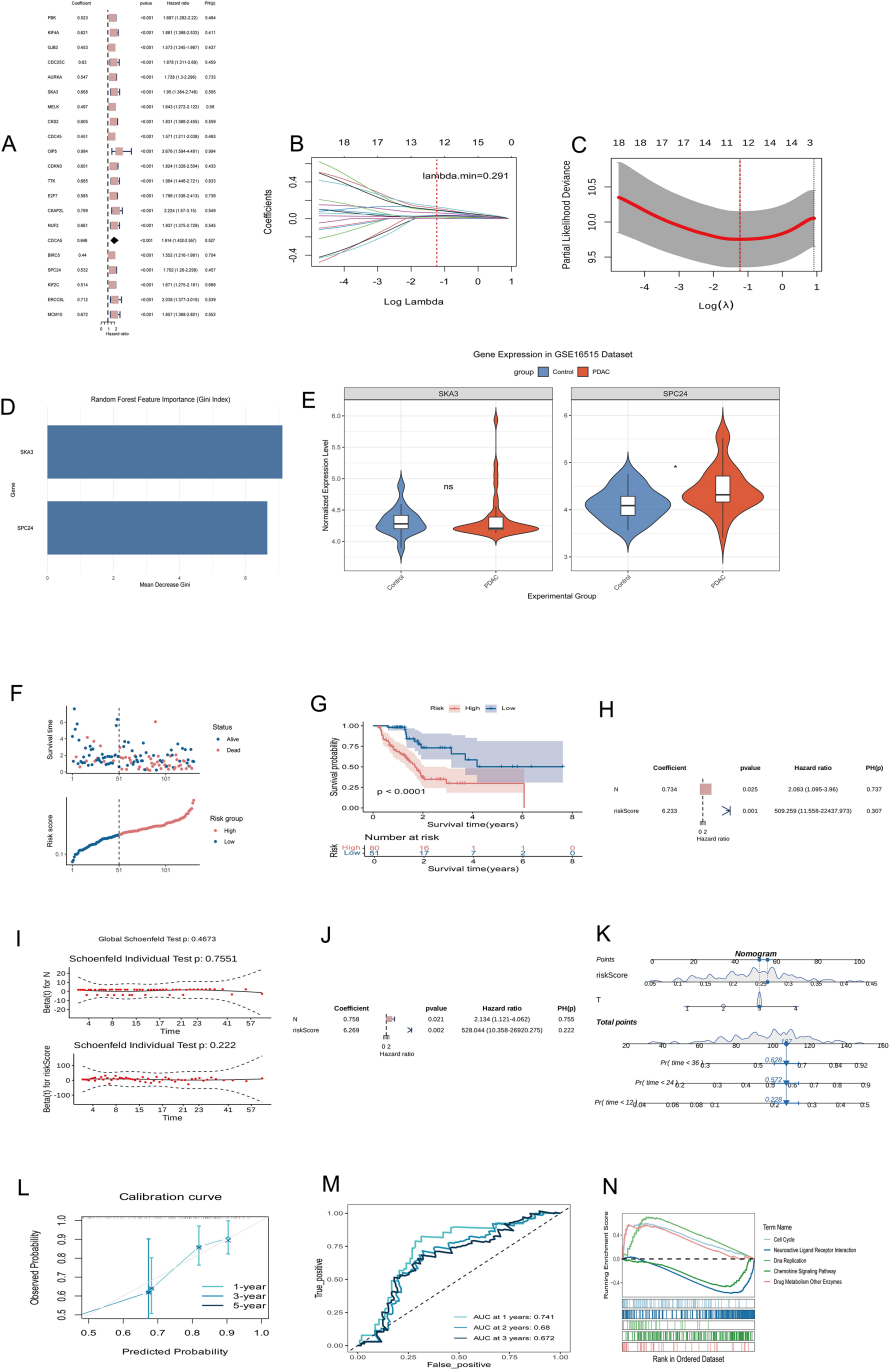
**(A)** is a one-way Cox regression forest plot, gene name, coefficient, p-value, HR value and PH hypothesis test p-value of one gene for each behavior, red represents HR>1, blue represents HR<1; **(B)** is a LASSO regression gene coefficient curve, abscissa represents lambda, ordinate represents gene coefficient, each curve represents a gene; **(B)** The right figure is the LASSO regression cross-validation error diagram, the abscissa represents lambda, and the ordinate represents the error size of the model; **(D)** The top two genes in random forest importance; **(E)** is the validation analysis of SKA3 and SPC24 using the external dataset GSE16515; The top of **(F)** is the survival status distribution chart, the abscissa represents each patient, the ordinate represents survival time, and the bottom is the risk score distribution chart, The abscissa represents each patient, the ordinate represents the risk score; **(G)** is the K-M curve, the abscissa represents the survival time, the ordinate represents the survival probability, and the lower table represents the number of survivors in the two groups at each time point; **(H)** is the one-way Cox regression and multi-way Cox regression forest plots, with variable names, coefficients, p-values, HR values and PH hypothesis test p-values for one variable in each behavior. In the figure, red represents HR>1, and blue represents HR<1; **(I)** is Schoenfeld for each variable over time Residual plot, the solid line is the smooth fitting line of scattered points, the dotted line represents the positive and negative two standard deviations of the fitting line, the abscissa is the survival time, the ordinate is the Schoenfeld residual; **(J)** is the multi-factor Cox regression analysis forest plot; **(K)** is a nomogram constructed based on Risk score and T stage. The horizontal axis of Risk score and T stage represents the risk score and T stage index of patients. Point represents the single score corresponding to each variable under different values. Total Point represents the single score corresponding to all variable values. The total score of the sum of the item scores, 1/2/3 year survival represents the survival probability of 1/2/3 years; **(L)** is the calibration curve to evaluate the accuracy of the nomogram model prediction, the horizontal axis and vertical axis represent the prediction probability and actual probability respectively, and the 3 broken lines represent the prediction performance of 1/2/3 years. The closer the broken line is to the diagonal, the closer the model prediction probability and actual probability are. **(M)** is ROC curve, abscissa and ordinate represent true positive and false positive rate respectively; **(N)**. KEGG enrichment analysis of SPC24 using GSEA shows the top 5 most significant signal pathways.

In order to further screen for genes related to tumor prognosis, a single-factor Cox regression analysis was first performed. As shown in **Figure 2A**, the results of single-factor Cox regression analysis show that multiple genes are related to survival, some of which are dangerous genes (HR>1) and some are protective genes (HR<1). Use the “forestplot” package to draw a forest map to display the results (**Figure 2A**).

In order to ensure the validity and reliability of the single-factor results, based on the single-factor results, use “cox. The “zph” function performs a PH hypothesis test, with P>0.05 as the threshold value, and finally multiple genes pass the test (**Figure 2A**), which are recorded as candidate prognostic genes for subsequent analysis. LASSO (Least absolute shrinkage and selection operator) analysis is a data mining method that adds penalty coefficients to multivariate linear regression, and continuously compresses the coefficients to reduce the dimension of the data and streamline the model, effectively avoiding collinearity and overfitting. Based on the results of the previous PH hypothesis test, LASSO regression analysis of candidate prognostic genes was used to further screen the candidate prognostic genes using the R package “glmnet”. Genes that are more important to the disease are compressed less, while genes that are less important are compressed into 0. After cross-verification, LASSO regression is obtained. There are two common graphs, one is the graph of the gene coefficient (left picture), and the other is the error graph of cross-verification (right picture). According to the LASSO regression results, 11 prognostic genes were finally screened, namely: PBK, SKA3, MELK, CKS2, OIP5, TTK, E2F7, CKAP2L, NUF2, CDCA5, SPC24 (**Figure 2B**). The values in parentheses are the LASSO coefficients of the corresponding genes. In order to further verify the importance of prognostic genes, a random forest algorithm was used for verification. The results are shown in **Figure 2D**. SKA3 and SPC24 are the top two genes in importance. In order to evaluate the expression of prognostic genes, SKA3 and SPC24 were verified and analyzed using the external data set GSE16515. The results are shown in **Figure 2E**. The expression of SPC24 in tumor samples was significantly increased.

### 3.7 Evaluation and verification of prognostic model

In order to evaluate the risk model composed of the SPC24 gene, the RiskScore formula was used in the TCGA training set to calculate the risk score of each patient. The patient sample was divided into high- and low-risk groups based on the best truncation value. Then a risk graph is drawn from low to high according to the patient’s risk score. As shown in **Figure 2F**, in the patient survival status distribution map (top), the red dots represent dead patients and the blue dots represent surviving patients; in the risk score distribution map (bottom), the red dots represent high-risk patients and the blue dots represent low-risk patients. The proportion of deaths among patients in the high-risk group was significantly higher than that of patients in the low-risk group.

In order to assess the survival differences between high and low risk groups, the R package “survival” is used to draw a K-M curve (Kaplan-Meier survival curve) based on the high and low risk groups. As shown in **Figure 2G**, the Wilcoxon test showed that there was a significant difference in survival rate between the two groups (P<0.05), p value<0.0001, and the survival rate of patients in the high-risk group is lower.

### 3.8 Independent prognosis analysis and construction of line chart

In order to further evaluate the survival of tumor patients based on independent prognostic factors, single-factor and multi-factor Cox regression analysis of risk scores and clinicopathological characteristics were carried out to construct a prognostic model to predict the OS survival of tumor patients. First, the risk scores and clinical features of tumor patients in the TCGA training set are integrated, and the R package “survival” is used to perform a single-factor Cox regression analysis of multiple variables of risk scores and clinical features. The screening criteria are HR ≠1, P value < 0.05, Use the R package “forestplot” to draw a forest map for visualization. As shown in **Figure 2H**, the single-factor Cox analysis results show the risk score, which is related to survival (P value < 0.05). In order to ensure the validity and reliability of the single-factor results, based on the single-factor results, use “cox. The “zph” function performs a PH hypothesis test, with P>0.05 as the threshold value, and the final risk score and N staging variables pass the test (**Figure 2H**). The Schoenfeld residual test (**Figure 2I**) was then used to evaluate the PH hypothesis, and the results showed that most variables did not violate the PH hypothesis (p-values were greater than 0.05), which provided a reliable basis for subsequent multi-factor Cox regression. Select these two significant variables for the next step of multi-factor Cox analysis. Use the R package “survival” to perform a multi-factor Cox analysis of the risk score of the 2 variables filtered out by the single-factor Cox. The filter criteria are HR ≠1, P value < 0.05. As shown in **Figure 2J**, the PH hypothesis test of the results of the multi-factor Cox regression analysis is performed. The P value of the two variables is >0.05, and the results of the multi-factor Cox regression analysis are reliable. Use the R package “forestplot” to draw a forest map for visualization. The analysis results are shown in **Figure 2J**. The risk score can be used as an independent prognostic factor (P value < 0.05). In order to further verify the predictive performance of the constructed risk model, based on the tumor samples of the TCGA training set and the independent prognostic indicators selected based on multivariate Cox regression analysis, a line chart was constructed using the “regplot” package to predict the survival rate of patients at different times (**Figure 2K**), and evaluated by drawing a calibration curve. The deviation between the predicted value of the column chart and the actual value (**Figure 2L**), the closer the slope of the calibration curve is to 1, the better the accuracy of the model’s prediction. **Figure 2M** shows the ROC curve, showing the prediction performance of the model in 1 year, 2 years and 3 years. The AUC values of the 3 curves are all greater than 0.6, and they have reached a higher level at several points in time, suggesting that the prognosis model has a reference predictive ability.

### 3.9 Gene set enrichment analysis

In order to explore the biological pathways of prognostic gene participation, in the TCGA training set, for tumor patients with OS survival information, tumor patients are first divided into high and low expression groups according to the optimal threshold of prognostic gene expression, and the R package “DESeq2” is used to analyze the differences between the high and low expression groups of each gene, and analyze the differences according to the differences. The results sorted all genes from large to small according to log2FC, and obtained a list of related genes corresponding to the high and low expression groups of each prognostic gene. After that, “c2. cp. kegg. v7.0. symbols. gmt” As a reference gene set, use the R package “clusterProfiler” for GSEA to explore the potential functions of prognostic genes, and use the R package “GseaVis” to show the enrichment results of prognostic genes (**Figure 2N**, p < 0.05, FDR < 0.25,|NES|>1).

### 3.10 Analysis of differences in immune microenvironment between high and low risk groups

**Figure 3:**
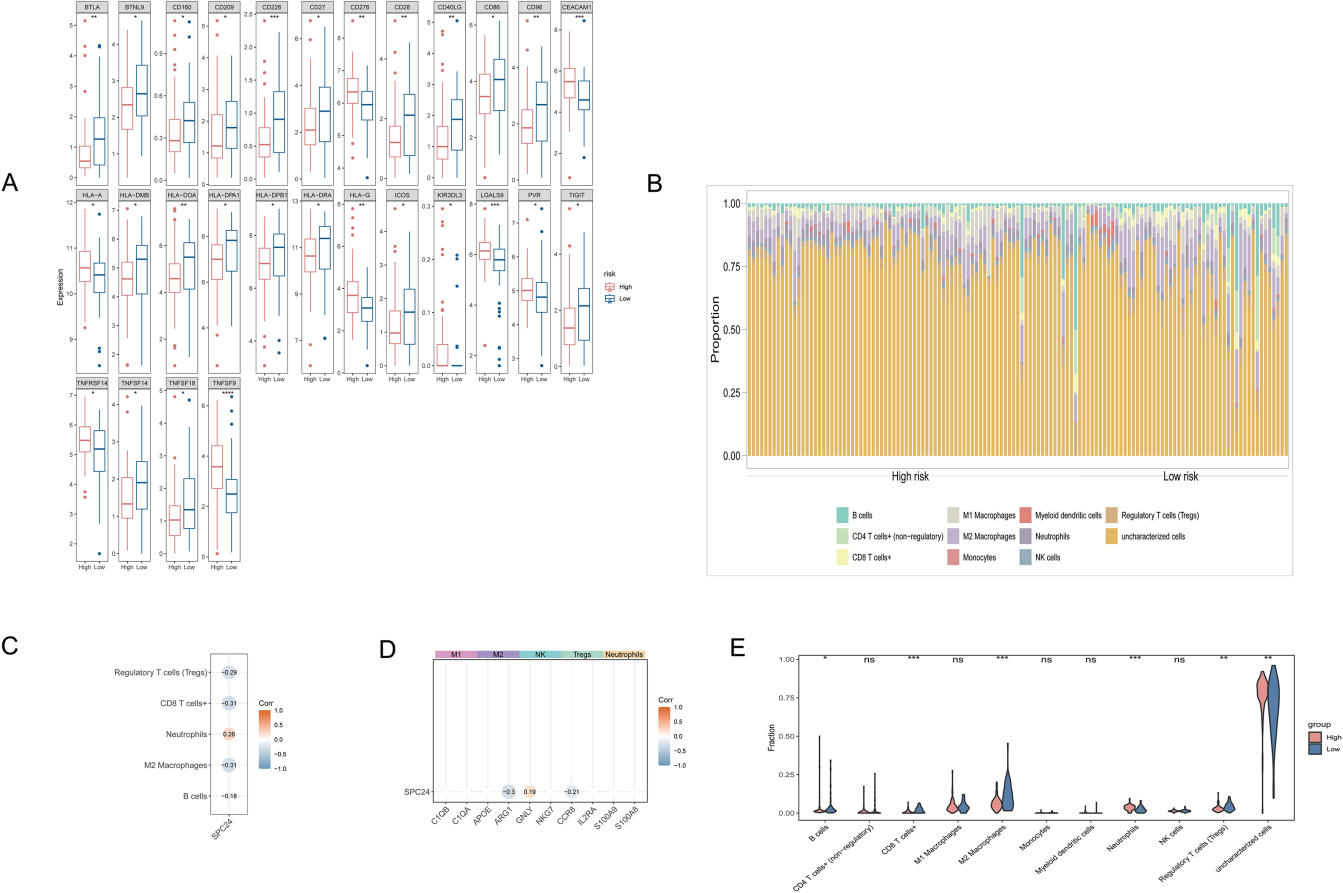
**(A)** is the expression difference of immune checkpoint genes in patients with high and low risk groups. The horizontal axis is sample grouping, and the vertical axis is gene expression;*** represents p <0.0001, ** represents p <0.001, * represents p <0.05, ns represents p>0.05. **(B)** is a histogram of immune infiltration. Each color in the histogram indicates a cell type. The horizontal axis is the sample and the vertical axis is the abundance of cell infiltration. **(C, D)** are correlation heat maps, color blocks in the heat map, orange for high correlation, blue for low correlation, showing only p<0.05 correlations. **(E)** is a violin plot of immune infiltration, with cell type on the horizontal axis and infiltration abundance on the vertical axis. **** represents p<0.0001, *** represents p<0.001, ** represents p<0.01, * represents p<0.05, ns represents p>0.05.

In order to explore the heterogeneity of the immune microenvironment between the high-risk group and the low-risk group, based on the TCGA training set data, the infiltration levels of 10 immune cells were quantified by the “quanTIseq” algorithm in the R package “immunedeconv”. The histogram of the immune infiltration stack (**Figure 3B**) shows that the proportion of uncharacterized cells in the two groups of samples is the highest (>60%), followed by regulatory T cells (Tregs) and M2 macrophages. Wilcoxon rank and test were further used to screen for different immune cells between the two groups (p<0.05). The above figure (**Figure 3E**) reveals that the proportion of M2 macrophages (p=0.003), neutrophils (p=0.015) and Tregs (p=0.021) infiltration in the high-risk group was significantly increased, while the abundance of CD8+T cells (p=0.028) and NK cells (p=0.042) was significantly reduced, Suggest the characteristics of the immunosuppressive microenvironment of the high-risk group.

### 3.11 Immune checkpoint gene expression and cell interaction network

The expression analysis of 79 immune checkpoint genes (ICGs) showed that 43 genes had significant differences between high and low risk groups (Wilcoxon test, p<0.05). The boxplot (**Figure 3A**) highlights key gene expression characteristics: HLA-DRA (p=1.2 e-5) and CD274 (PD-L1, p=0.003) are highly expressed in the low-risk group; TNFSF9 (p=0.007) and LAG3 (p=0.012) are significantly upregulated in the high-risk group. The correlation heat map (**Figure 3C**) shows the interaction between immune cells: M2 macrophages are strongly positively correlated with Tregs (Cor=0.62, p=0.001), while CD8+T cells are negatively correlated with Tregs (Cor= -0.41, p=0.013). In addition, the expression of the prognostic gene SPC24 was positively correlated with M2 macrophages (Cor=0.38, p=0.002) and neutrophils (Cor=0.29, p=0.018), but was significantly negatively correlated with NK cells (Cor= -0.25, p=0.032) (**Figure 3D**). In order to understand the differences between different immune cells in the samples of the high-risk group and the low-risk group, in the TCGA training set, use the Wilcoxon rank and test to compare the differences in the infiltration levels of various immune cells between the samples of the high-risk group and the low-risk group (P < 0.05), obtain different immune cells, use the R package “ggplot2”Draw a violin diagram to show (**Figure 3E**). The results showed that B cells, CD8+T cells, M2 macrophages, neutrophils, Treg cells and uncharacterized cells had infiltration differences between samples in the high-risk group and the low-risk group(p < 0.05).

### 3.12 Analysis of single-cell sequencing data

#### 3.12.1 Identification of highly variable genes

**Figure 4:**
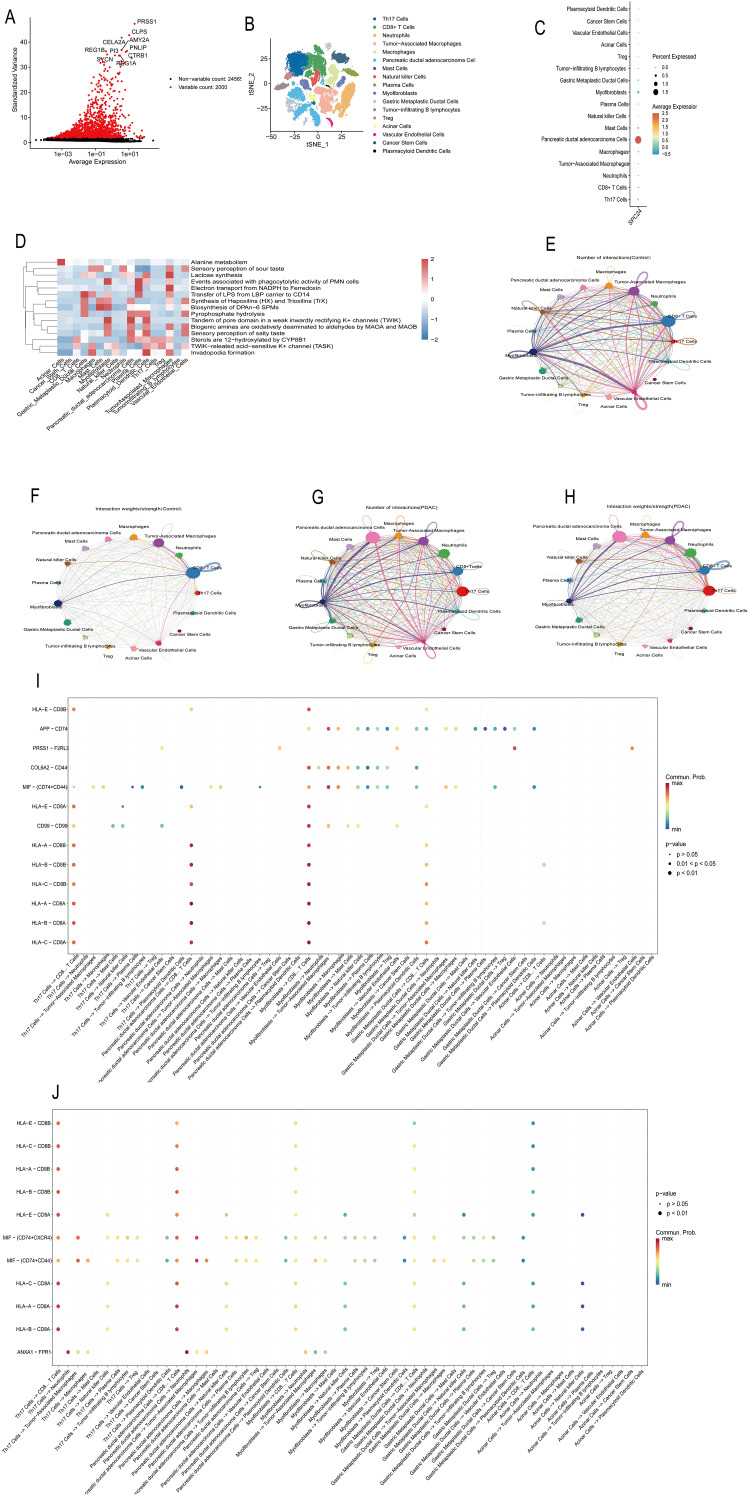
**(A)** Identification of hypervariable genes, abscissa represents expression mean, ordinate represents variance, each point represents a gene, wherein red represents identified hypervariable genes, black represents excluded genes; **(B)** TSNE cluster diagram of 17 cell types, abscissa represents relative positions of two dimensions after cell dimension reduction, and each point in the diagram represents a cell; **(C)** SPC24 gene expression bubble diagram, abscissa is SPC24 gene, ordinate is different cell types, the redder the bubble color, the higher the expression of SPC24 gene in the cell, the size of the bubble represents the proportion of SPC24 gene expression in a type of cell; **(D)** Metabolic pathway enrichment results at the single-cell level, with the x-axis representing cell names and the y-axis representing pathway names, red indicating upregulation, and blue indicating downregulation; **(E)** is the number of interactions between cell subsets in the control group, the arrow represents the direction, and the line thickness represents the number; **(F)** is the weight of interaction between cell subsets in control group, arrow represents direction, line thickness represents weight; **(G)** is the number of interaction between cell subsets in PDAC group, arrow represents direction, line thickness represents number; **(H)** is the weight of interaction between cell subsets in PDAC group, arrow represents direction, line thickness represents weight; **(I)** is a bubble plot of ligand pairs interacting among cell subsets in the control group, the horizontal axis represents the interaction direction between different cells, the vertical axis represents specific ligand pairs, different colors of dots represent communication probability, and the size of dots represents P value of communication significance; **(J)** is a bubble plot of ligand receptor pairs interacting among cell subsets in PDAC group. The horizontal axis represents the interaction direction between different cells, the vertical axis represents specific ligand receptor pairs, the different colors of dots represent communication probability, and the size of dots represents P value of communication significance.

In order to identify genes that play an important role in the development of tumorigenesis, we first standardized the single-cell sequencing data, and used analysis of variance to screen out the top 2,000 genes with the largest expression level variation, defined as highly variable genes, the results are shown in **Figure 4A**. Through the LablePoints function, we marked the top 10 genes with the greatest expression level variation, which may play a key role in the heterogeneity and functional regulation of tumor cells.

#### 3.12.2 Cell type annotation and clustering

In order to analyze the cell composition of the tumor microenvironment, we performed dimensional-reducing clustering analysis of single-cell data. First, the high-dimensional data is reduced to 20 principal components by the PCA method, and then the TSNE algorithm is used for nonlinear dimensionality reduction, and the cells are divided into different cell clusters according to the gene expression profile of the cells. The results showed that there are multiple cell types in the tumor microenvironment (**Figure 4B**).

In order to further determine the cell type of each cell cluster, we annotated the cell cluster using known cell marker genes based on published literature and the CellMarker database. Finally, the following 17 cell types were identified: Th17 Cells, CD8+TCells, Neutrophils, Tumor-associated Macrophages, Macrophages, Pancreatic ductal adenocarcinoma Cells, Mast Cells, Natural killer cells, Plasma Cells, Myofibroblasts, Gastrointestinal Metaplastic ductal cells, Tumor-infiltrating B lymphocytes, Treg, Acinar Cells, Vascular Endothelial Cells, Cancer Stem Cells, Plasmacytoid Dendritic Cells. We used bubble diagrams to show the expression of marker genes in different cell types (**Figure 4C**) and verified the accuracy of cell type annotations. Meanwhile, we performed metabolic pathway enrichment analysis on cells from each subpopulation at the single-cell level, and Alanine metabolism showed a high positive correlation with Acinar cells, while Lactose synthesis showed a high positive correlation with Tumor-associated Macrophages (**Figure 4D**).

#### 3.12.3 Analysis of cell communication and preliminary explanation of immune escape mechanism

In order to explore the interaction between cells in the tumor microenvironment, we used CellChat to analyze the single-cell sequencing data of the Control group and the PDAC group, focusing on changes in ligand-receptor interaction. The results showed that compared with the Control group, the communication mode between cells in the PDAC group changed significantly (**Figures 4E–J**). In the control group, the interaction between endothelial cells (Vascular endothelial cells) and macrophages through Jam2-F11r showed a strong probability of communication. However, in the PDAC group, this interaction was significantly weakened, suggesting that the tumor may affect the production of tumor blood vessels and the recruitment of immune cells by changing the communication between endothelial cells and macrophages. It is worth noting that the interaction of macrophage migration inhibitory factor (MIF) and its receptors CD74, CXCR4, and CD44 was significantly enhanced in the PDAC group. Specifically, the interaction of MIF-CD74 between endothelial cells and macrophages and tumor-associated macrophages showed an enhanced trend. MIF is a multifunctional cytokine that is involved in regulating the function of immune cells in the tumor microenvironment. Activation of the MIF-CD74 signaling pathway may lead to the recruitment and activation of immunosuppressive cells (such as tumor-associated macrophages) in the tumor microenvironment, thereby inhibiting the anti-tumor immune response of T cells and promoting tumor immune escape.

In addition, we observed that the HLA-A-CD8A/B/C coordination receptor pair also showed an enhanced trend in PDAC samples. HLA-A is an MHC class I molecule that is responsible for presenting tumor antigen to CD8+T cells and activating their killing function. However, tumor cells may inhibit the activity of T cells by upregulating immune checkpoint molecules (such as PD-L1) and other mechanisms, thereby evading immune surveillance. Therefore, the enhancement of HLA-A-CD8A/B/C interaction may reflect that tumor cells are trying to activate T cells, but at the same time inhibit T cell function through other mechanisms, and ultimately realize the complex process of immune escape.

In summary, the results of our single-cell sequencing analysis reveal the complex cell composition and intercellular interaction patterns of the tumor microenvironment. PDAC cells may regulate cell interaction in the tumor microenvironment through a variety of mechanisms, thereby promoting immune escape. The MIF-CD74 signaling pathway and the interaction of HLA-A-CD8A/B/C may be potential therapeutic targets and deserve further research.

### 3.13 SPC24 promotes the proliferation, colony formation and migration of PAAD cells *in vitro*, as well as tumor growth *in vivo*

**Figure 5:**
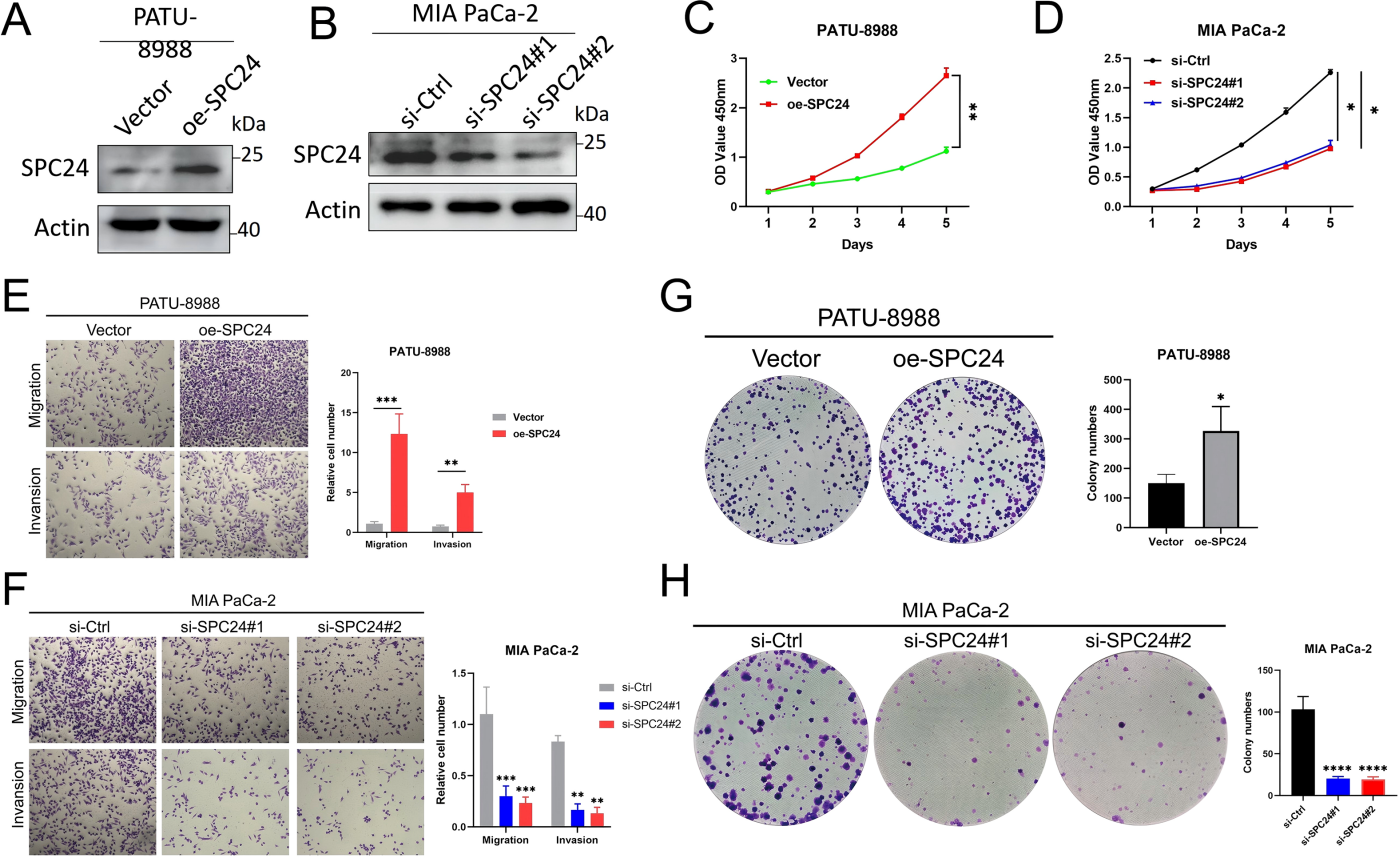
The oncogenic function of SPC24 in PAAD cells **(A, B)** Western blotting results of PAAD cells transfected with empty or SPC24-overexpressing plasmids, and with control siRNA or si-SPC24. **(C, D)** CCK8 assay result of PAAD cells transfected with empty or SPC24-overexpressing plasmids, and with control siRNA or si-SPC24. **(E, F)** Transwell assay result of PAAD cells transfected with empty or SPC24-overexpressing plasmids, and with control siRNA or si-SPC24. **(G, H)** Colony-forming assay result of PAAD cells transfected with empty or SPC24-overexpressing plasmids, and with control siRNA or si-SPC24. * represents p<0.05, ** represents p<0.01, *** represents p<0.001, **** represents p<0.0001.

**Figure 6:**
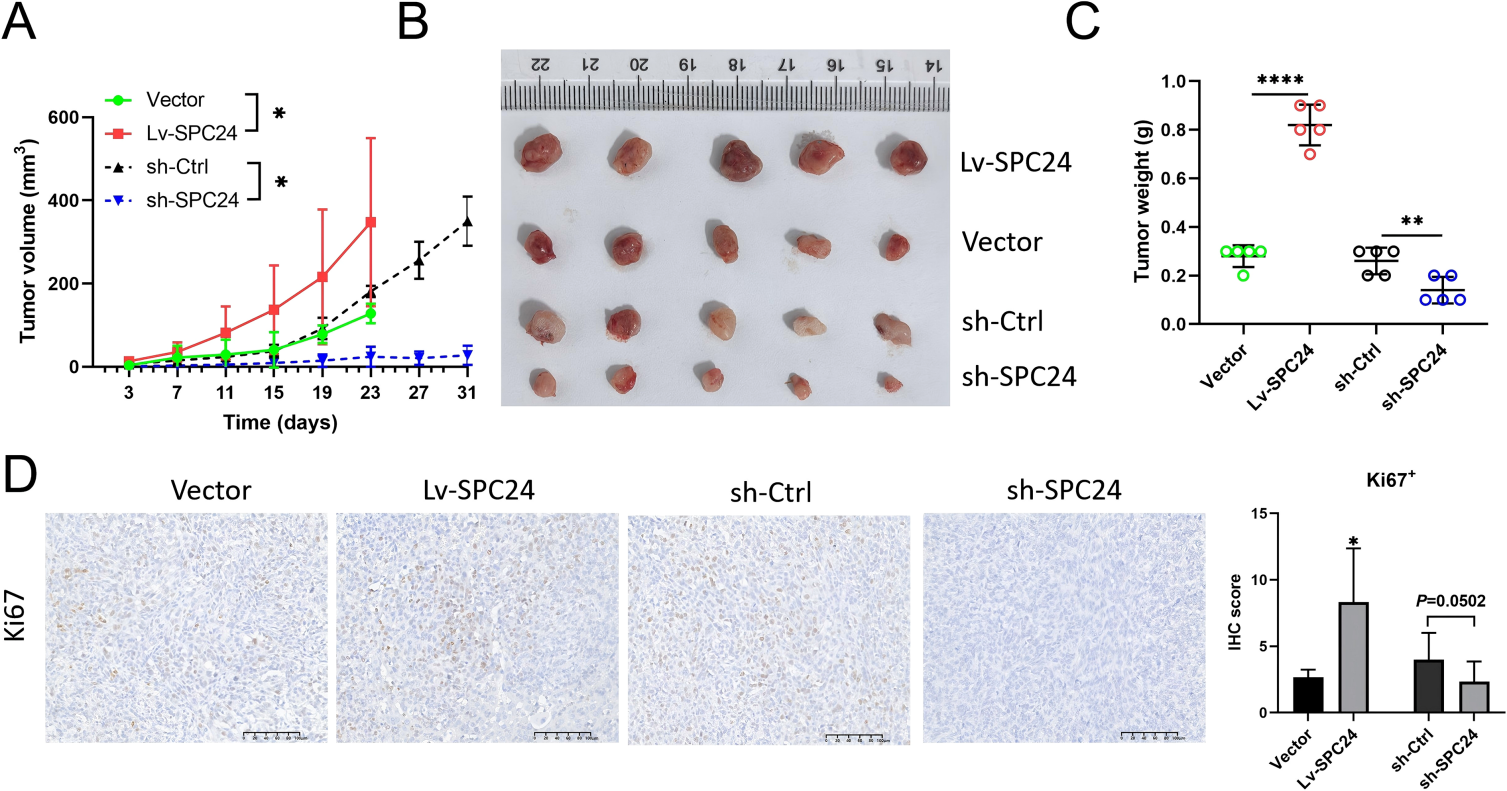
SPC24 positively regulates PAAD growth *in vivo*. **(A–C)** Overexpression of SPC24 promotes PAAD growth, while knockdown of SPC24 inhibits PAAD growth. **(D)** PAAD tissues in SPC24-overexpression group exhibited higher proliferative activity, whereas lower proliferative activity was detected in SPC24-knockdown group. * represents p<0.05, ** represents p<0.01, **** represents p<0.0001.

In order to determine the role of SPC24 in regulating PAAD cell behavior, we constructed PAAD cell lines with elevated or decreased SPC24 expression levels, and verified their successful operation through Western blot analysis (**Figures 5A, B**). CCK8 experiments showed that when SPC24 is overexpressed, PAAD cell proliferation increases, while proliferation slows down when SPC24 is knocked down (**Figures 5C, D**). In addition, colony formation experiments have shown that the ability of PAAD cells to form clones is enhanced when the expression level of SPC24 is increased, while reducing the level of SPC24 weakens this ability (**Figures 5E, F**). Further, Transwell experiments have shown that overexpression of SPC24 leads to enhanced migration ability of PAAD cells, while inhibition of SPC24 weakens this migration ability (**Figures 5G, H**). These *in vitro* results show that SPC24 plays a key role in enhancing the proliferation ability, colony formation ability and migration ability of PAAD cells. In addition to *in vitro* experiments, we also established a naked mouse xenograft PAAD model to study the role of SPC24 in the progress of PAAD *in vivo*. Overexpression or knockdown of SPC24 in PAAD cells and control group cells were injected subcutaneously into naked mice, and tumors were collected three weeks after injection. As shown in **Figures 6A, B**, the tumor volume of the SPC24 overexpression group and the SPC24 knockdown group were significantly larger and smaller than that of the control group, respectively. Consistently, compared with the tumors in the control group, the tumor weight of the SPC24 overexpression group and the SPC24 knockdown group were higher and lower, respectively (**Figure 6C**). In addition, higher proliferative activity was observed in the tumor tissue of the SPC24 overexpression group, while lower proliferative activity was detected in the tumor tissue of the SPC24 knockdown group (**Figure 6D**). In summary, these results confirm the carcinogenic role of SPC24 in the advancement of PAAD.

## 4 Discussion

The aggressive biology of pancreatic ductal adenocarcinoma (PDAC) interacts with the complex tumor microenvironment (TME), resulting in treatment resistance and poor prognosis. By integrating multi-omics data and functional experiments, this study systematically revealed the key mechanism of SPC24 in PDAC and its clinical transformation potential for the first time, providing a new theoretical basis for the accurate diagnosis and treatment of PDAC. The prognostic value of SPC24, molecular mechanisms driving tumor progression, immune microenvironment regulation and potential therapeutic significance are discussed below.

### 4.1 SPC24 as a novel prognostic molecular marker for PDAC

In this study, TCGA, ICGC, and GEO multi-cohort analysis demonstrated that SPC24 mRNA and protein were significantly overexpressed in PDAC tissues (log2FC =2.1, p<0.001) and independently negatively correlated with overall survival (OS) and progression-free survival (PFS)(HR = 2.15, p=0.009). It is worth noting that the nomogram constructed by the risk score model of SPC24 (AUC = 0.78) combined with clinical pathological characteristics shows good predictive performance (calibration curve slope>0.9), suggesting its clinical application value in prognostic stratification. This result is consistent with the role of SPC24 in gastric cancer and lung cancer, but its prognostic association in PDAC is clear here for the first time (18–20). Further single-cell sequencing analysis revealed that SPC24 is specifically overexpressed in PDAC malignant cell subsets, and the metabolic characteristics of this subset are closely related to invasive phenotypes: 1) Invadopodia formation regulation: Malignant cells overexpressing SPC24 exhibit significant Invadopodia activity Promotes extracellular matrix (ECM) degradation and local invasion (via upregulation of matrix metalloproteinases MMP-2/9 and integrin β1 signaling pathways), consistent with the mechanism by which cancer cells rely on Invadodia to break through the basement membrane and enter the circulatory system during PDAC metastasis;2) Metabolic reprogramming drives invasion: Metabolic analysis revealed abnormal activation of TWIK-related potassium channels (e.g. TASK-3) in this subset, resulting in changes in intracellular pH homeostasis, which in turn drives glycolytic reprogramming through activation of HIF-1α signaling (Warburg effect); This metabolic adaptation not only supports the energy requirements of cancer cells, but also promotes TME acidification through lactic acid accumulation, further activating MMPs and enhancing invasion; 3) Clinical treatment implications: The association of SPC24 with Invadopodia formation and metabolic abnormalities suggests that targeting SPC24 or its downstream effector molecules (e.g. MMPs or TWIK channels) may inhibit PDAC metastasis, e.g., combining Invadopodia inhibitors (e.g. TKS5 antagonists) with metabolic modulators (metformin) may break through current treatment bottlenecks (21–23). The formation of Invadopodia depends on the activation of Src kinase/Cdc42 signaling axis, and SPC24 may enhance the ability of cytoskeleton remodeling by regulating this pathway. Meanwhile, the ion current mediated by TWIK channel may affect the cell membrane potential, and then regulate the endocytosis cycle of growth factor receptors such as EGFR, forming a positive feedback to promote invasion (21).

Notably, high expression of SPC24 was significantly associated with tumor genomic instability (e.g., activation of nonhomologous terminal junction pathways, PAL = 1.82e-11). As a core component of the γ-tubulin complex (γ-TuRC), SPC24 may affect the accuracy of chromosome separation by regulating microtubule nucleation dynamics (24). Overexpression of SPC24 in PDAC cells may lead to spindle assembly errors, resulting in chromosome aneuploidy and copy number variation (CNA), consistent with the high mutation load characteristic of PDAC (25–28). Therefore, SPC24 is not only a prognostic marker, but its expression level may directly reflect genomic instability of tumors and provide clues for therapeutic strategies targeting DNA damage repair (DDR) pathway.

### 4.2 Molecular mechanism of PDAC driven by SPC24

Overexpression of SPC24 significantly enhanced proliferation, migration and subcutaneous tumorigenesis of PDAC cells, while knockdown of SPC24 reversed the phenotype. Further mechanistic studies suggest that SPC24 drives PDAC progression through the following multidimensional mechanisms:

(1) Cell cycle disorder and mitotic abnormalities.

GO and KEGG enrichment analysis showed that SPC24 co-expressed genes were significantly enriched in chromosome segregation (NES = 2.8) and spindle assembly (NES = 2.5). We found that overexpression of SPC24 promotes genomic instability and clonal evolution of PDAC through the following mechanisms: 1)Aurora B kinase activity is abnormally upregulated: overexpression of SPC24 significantly enhances Aurora B kinase activity (confirmed by elevated levels of autophosphorylation at its Thr232 locus), resulting in centrosome amplification (manifested by increased γ-tubulin aggregation) and multipolar spindle formation; 2) Increased Aurora B activity exacerbates chromosome missegregation through two pathways: centrosome amplification: Increased number of abnormal centrosomes leads to spindle multipolarization (e.g., tripolar or quadrupolar), resulting in unequal chromosome distribution, Aurora B overexpression impairs its ability to correct kineto-microtubule misconnections (e.g., decreasing BubR1 phosphorylation efficiency), increasing the risk of chromosome lag or missegregation;3)CIN driven clonal evolution acceleration: Although cells with multipolar spindle formation can complete division through “pseudo-bipolarization”(centrosome clustering), residual chromosome mismatches (e.g., micronuclei or lagging chromosomes) continue to accumulate copy number variation (CNA), which is highly consistent with PDAC subclonal heterogeneity in single cell sequencing (29–31). In addition, SPC24 shortens mitotic checkpoint duration by activating the PLK1-CDC20 axis, allowing cells to advance into anaphase before DNA damage is repaired, thereby accumulating oncogenic mutations.

(2) Metabolic reprogramming and microenvironment acidification.

Single-cell metabolic pathway analysis reveals glycolysis in PDAC cells with high expression of SPC24 (LDHA, PKM2) and glutamine breakdown (GLS) pathways were significantly activated (NES>2.0) and lactate secretion increased 3.2 fold. This metabolic phenotype not only provides energy for tumor proliferation, but also inhibits CD8 + T cell activity (IFN-γ secretion decreased by 62%) and dendritic cell maturation (CD80/CD86 expression decreased by 45%) by acidifying TME (pH decreased by 0.5 unit), forming an immunosuppressive niche (32–34).

(3) Microbe-tumor interaction synergistically promotes cancer.

Microbiome analysis revealed clostridium nucleatum in the high expression group of SPC24 (Fusobacteria nucleatum) abundance significantly increased (log2FC =3.8), which activates the NF-κB signaling pathway by binding to Toll-like receptor 4 (TLR4) on PDAC cell surfaces This positive feedback loop may explain the persistent association between SPC24 and tumor progression. In addition, Clostridium nucleatum promotes tumor cell invasion by secreting virulence factor FadA (1.7-fold increase in Transwell migration ability), suggesting that microbial intervention may enhance the efficacy of SPC24 targeted therapy (35, 36).

### 4.3 Mechanism of SPC24 remodeling immunosuppressive microenvironment

Through single-cell sequencing and CellChat interaction analysis, this study reveals the key pathways through which SPC24 drives immune escape by regulating ligand-receptor networks:

(1) TAMs polarization and immunosuppression.

In this study, PDAC cells overexpressing SPC24 bind to CD44 on TAMs by secreting COL 6A2, which may be related to activation of PI 3K-AKT-mTOR pathway and induction of TAMs to M2 phenotype polarization. M2 TAMs further secrete IL-10 and TGF-β1, inhibit CD8 + T cell toxicity and promote Treg expansion (37).

(2) CD99 mediated physical barrier formation.

SPC24 promotes CD99 expression by activating Notch/Hes1 pathway in myofibroblasts and forms a dense extracellular matrix barrier through CD99-CD99 isotype interaction, which significantly inhibits CD8 + T cell infiltration. CD99 also enhances myofibroblast-tumor cell adhesion through integrin β1 signaling, promotes mechanical stress transmission and activates profibrotic pathways (38).

(3) Metabolic competition and immune exhaustion.

Lactic acid accumulation driven by SPC24 downregulates glycolytic capacity and killing function by inhibiting mTORC1 activity in CD8 + T cells. At the same time, lactic acid induces PD-1 expression in CD8 + T cells by stabilizing HIF-1α, leading to terminal exhaustion phenotype (39, 40).

### 4.4 Clinical implications and future directions

The findings of this study provide a multidimensional strategy for precision treatment of pancreatic ductal adenocarcinoma (PDAC):(1) Prognostic stratification and dynamic monitoring: a risk score model based on SPC24 expression levels (AUC = 0.78) can effectively discriminate high-risk patients (3-year survival <15%) and guide intensive treatment (e.g. FOLFIRINOX regimen combined with immune checkpoint inhibitors) (41, 42). Notably, integration of matrix-related markers such as SPARC (Secreted Protein Acidic and Cystine Rich) may further enhance the predictive accuracy of the model, as it can influence PDAC progression by regulating angiogenesis and hypoxic microenvironments. In addition, SPC24 copy number variations in circulating tumor DNA (ctDNA) combined with liquid biopsy techniques such as CA19–9 dynamic monitoring may be used as a noninvasive monitoring tool with a 23% increase in sensitivity over a single marker (41, 42).

(2) Therapeutic strategies targeting SPC24.

The development of small molecule inhibitors of γ-TuRC binding domain targeting SPC24, such as AI-designed peptide antagonists, can act by a dual mechanism: directly inducing mitotic disaster in tumor cells (2.5-fold increase in apoptosis rate) while reversing the immunosuppressive microenvironment (42% reduction in Treg ratio and 1.8-fold increase in CD8 + T cell infiltration). Preclinical studies have shown that SPC24 siRNA nanoparticles in combination with PD-1 antibody significantly inhibit tumor growth (78% reduction in volume) and that combination with antiangiogenic drugs such as bevacizumab further improves chemotherapy drug delivery efficiency (3.1-fold increase in gemcitabine concentration in tumors) (41, 42). Furthermore, targeting pathways downstream of SPC24 (e.g. COL6A2-CD44 axis or YAP/TAZ signaling) may enhance efficacy by inhibiting polarization and collagen deposition of tumor-associated macrophages (TAMs).

(3) Microbial intervention adjuvant therapy.

Elimination of Fusarium nucleatum by antibiotics or supplementation with probiotics such as Lactobacillus may attenuate the carcinogenic effects of SPC24. Animal experiments showed that metronidazole combined with SPC24 inhibitor reduced tumor burden by 64% and significantly improved fibrotic microenvironment (collagen area reduced by 52%) (41, 42). It is suggested that microbial-metabolic axis intervention may be an important auxiliary means for PDAC targeted therapy.

Future research directions should focus on: 1) functional heterogeneity of SPC24: defining its molecular subtypes in PDAC (e.g. basal-like *vs*. classical), especially how genomic instability and immune escape are synergistically driven in the context of KRAS mutations (e.g. G12D);2) diagnostic tool development: design of SPC24-specific PET probes (e.g. radiolabeled ligand based on its gamma-TuRC binding properties) to enable dynamic monitoring of efficacy;3) Combination therapy optimization: explore synergistic effects of SPC24-targeted therapy with matrix remodeling strategies (e.g. Hedgehog pathway inhibitors) or metabolic interventions (e.g. LDHA inhibitors) to overcome the high heterogeneity and treatment resistance of PDAC (41, 42).

## 5 Conclusion

This study is the first to systematically elucidate the multidimensional mechanism of action of SPC24 in PDAC: it promotes tumor progression and treatment resistance by driving mitotic abnormalities, metabolic reprogramming and immune microenvironment remodeling. SPC24 can be used as a prognostic marker and potential therapeutic target, and combination therapies designed for it (such as inhibitors + immunotherapy + microbial regulation) are expected to break through the therapeutic bottleneck of PDAC. The regulatory network and translational applications of SPC24 need to be further explored to improve patient survival.

## Data Availability

The original contributions presented in the study are included in the article/supplementary material. Further inquiries can be directed to the corresponding author.
